# Impact of sensitization and ABO blood types on the opportunity of deceased-donor kidney transplantation with prolonged waiting time

**DOI:** 10.1038/s41598-024-53157-2

**Published:** 2024-02-01

**Authors:** Jin Hyeog Lee, Tai Yeon Koo, Jung Eun Lee, Kook Hwan Oh, Beom Seok Kim, Jaeseok Yang

**Affiliations:** 1 Division of Nephrology, Department of Internal Medicine, Yonsei University College of Medicine, 50-1 Yonsei-Ro, Seodaemun-Gu, Seoul, 03722 Republic of Korea; 2 Division of Nephrology, Department of Internal Medicine, Korea University Anam Hospital, Seoul, Republic of Korea; 3 Division of Nephrology, Department of Internal Medicine, Yongin Severance Hospital, Yongin, Republic of Korea; 4 Division of Nephrology, Department of Internal Medicine, Seoul National University College of Medicine, Seoul, Republic of Korea

**Keywords:** Nephrology, Risk factors

## Abstract

The waiting time to deceased-donor kidney transplantation (DDKT) is long in Asian countries. We investigated the impact of sensitization and ABO blood type (ABO) on DDKT opportunity using two Korean cohorts: a hospital cohort from two centers and a national database. The impact of panel reactive antibody (PRA) based on the maximal PRA% and ABO on DDKT accessibility was analyzed using a competing risks regression model. In the hospital cohort (n = 4722), 88.2%, 8.7%, and 3.1% of patients belonged to < 80%, 80–99%, and ≥ 99% PRA groups, respectively, and 61.1%, 11.6%, and 27.3% belonged to A or B, AB, and O blood types, respectively. When PRA and ABO were combined, PRA < 80%/A or B and 80 ≤ PRA < 99%/AB had fewer DDKT opportunities (median, 12 years; subdistribution hazard ratio [sHR], 0.71) compared with PRA < 80%/AB (median, 11 years). Also, PRA < 80%/O, 80 ≤ PRA < 99%/A or B, and PRA ≥ 99%/AB had a much lower DDKT opportunity (median, 13 years; sHR, 0.49). Furthermore, 80 ≤ PRA < 99%/O and PRA ≥ 99%/non-AB had the lowest DDKT opportunity (sHR, 0.28). We found similar results in the national cohort (n = 18,974). In conclusion, an integrated priority system for PRA and ABO is needed to reduce the inequity in DDKT opportunities, particularly in areas with prolonged waiting times.

## Introduction

Kidney transplantation is the best treatment option for patients with end stage renal disease (ESRD)^1–3^. Deceased donation rates have been much lower in Asian countries than in Western countries^4^, whereas the recent ESRD incidence is higher in Asian countries than in Western countries^5^. Therefore, donor organ shortages are more severe and the waiting time for deceased-donor kidney transplantation (DDKT) is much longer in Asian countries^6^.

In patients on the waiting list for DDKT, sensitization to human leukocyte antigen (HLA) is a major barrier to DDKT allocation^7,8^. By assessing the reactivity of a waitlist patients’ serum with HLA antigens on beads, the panel reactive antibodies (PRA) are reported as a percentage based on the frequency of positive HLA antigens in the estimated donor pool, and the overall probability of recipient mismatching for donor pools is approximated. PRAs indicate the sensitization status of waitlisted patients^9^. Prioritization of DDKT candidates with higher PRA has been adopted in many kidney allocation systems, including the Unites States (US), United Kingdom (UK), Eurotransplant, Australia and New Zealand, to provide a fair DDKT opportunity and maintain an equity principle^10–12^.

The ABO blood type also induce inequity in DDKT opportunity^13^ due to the biological ABO blood type compatibility between the donors and recipients. O-type candidates are only biologically compatible with O-type donors, which limits their potential donor pool. In contrast, AB-type candidates are biologically compatible with A, B, and O donors, expanding their potential donor pool.

In the Korean kidney allocation system, adult waitlisted patients are prioritized based on HLA full match. Among waitlisted patients with zero HLA mismatch, deceased donor kidneys are allocated to patients with identical ABO blood types. When there is no candidate with the identical blood types, kidneys are allocated to waitlisted patients with compatible blood types. In cases of HLA mismatches, allocation is determined through a point system that consists of the degree of HLA matching (0–2 points), duration on the waiting (1 point for each year of wait), history of kidney transplantation or repeated, positive cross-match test results (2 points), and personal or familial history of organ donation (2–4 points). There is no additional point according to ABO blood types or PRA levels.

Sensitization might have decreased DDKT accessibility more seriously in Asian countries, including Korea, which have a longer waiting time than Western countries. Yet, the impact of sensitization on DDKT waiting period in Asian countries has not been studied in a nationwide cohort. Therefore, we aimed to investigate the impact of sensitization and ABO blood types on DDKT opportunities in a Korean nationwide database as well as a hospital-based database.

## Patients and methods

### Patients

Data from two cohorts were analyzed in this study. First, the hospital cohort comprised 5322 waitlisted patients from Severance Hospital and Seoul National University Hospital between February 15, 2000 and July 31, 2021. Of those, 4722 patients were included in the final analysis after excluding 133 patients ≤ 18 years who received additional points in the current Korean kidney allocation scheme and 477 patients without PRA data (Supplementary Fig. S1). Second, the national cohort was retrieved from the Korean Organ Network for Organ Sharing (KONOS) database between January 1, 2000 and December 31, 2018. A total of 18,974 patients were included in the study from a total of 35,859 patients; 106 patients ≤ 18 years and 16,779 without PRA data were excluded (Supplementary Fig. S1).

This study was performed in accordance with the 2000 Declaration of Helsinki^14^ and the Declaration of Istanbul 2008^15^, and was approved by the Institutional Review Board of Severance Hospital (4-2023-0244) and Seoul National University Hospital (H-2304-061-1421). Informed consent was waived by the Institutional Review Board of Severance Hospital (4-2023-0244) and Seoul National University Hospital (H-2304-061-1421) owing to the retrospective nature of the study, which involved medical records without identifiable patient information.

### Data collection

Data from the hospital cohort were obtained from the electronic medical records of Severance Hospital and Seoul National University Hospital. National cohort data were obtained from the KONOS database. Clinical information, such as age, sex, ABO blood type, and diabetes mellitus status were extracted. The primary outcome was the number of waitlisted patients’ who underwent DDKT.

### PRA information

In the hospital cohort, PRA was assessed using LABScreen single-antigen assays, identification assays (One Lambda Inc., Canoga Park, CA, USA), LIFECODES single-antigen assay, or identification assays (Immunocor Inc., Norcross, GA, USA). Maximum PRA values in percentages (max PRA%) among class I and II PRA values in PRA identification assays were used before single-antigen bead assays were introduced, and we then used higher values (%) among class I and II calculated PRA (cPRA) in the single-antigen assays were used after single-antigen bead assays were introduced. In the national cohort, most PRA data were collected as positive or negative instead of as a specific percentage of PRA; therefore, these qualitative PRA results were used in the analysis. We defined a negative PRA as having a value of 0% for both PRA class I and class II. Conversely, we defined a positive PRA as a case where either class I or class II showed a PRA value greater than 0%. Waitlisted patients were categorized into three PRA groups according to the max PRA% as follows: low (PRA < 80%), intermediate (80 ≤ PRA < 99%), and high (PRA ≥ 99%) in hospital cohort. They were also categorized into positive and negative PRA groups in both hospital and national cohort.

### Statistical analyses

Comparisons of clinical characteristics between the PRA groups were performed using the Mann–Whitney U test or Kruskal–Wallis test for continuous variables. For categorical variables, the chi-squared test or Fisher’s exact test was used, as appropriate. Continuous variables were presented as medians (interquartile range [IQR]), and categorical variables were presented as absolute numbers (percentages). Kaplan–Meier survival analysis was used to assess the cumulative DDKT rates, and the log–rank test was used to compare DDKT rates between the PRA groups. The independent association of PRA groups or ABO bloody types with accessibility to DDKT was analyzed using Fine and Gray competing risks regression models to estimate the subdistribution hazard ratio (sHR) of DDKT, accounting for death while on the waiting list as a competing risk^16^. We reported the sHR with a 95% confidence interval (CI). The multivariate model for PRA was adjusted for age, sex, baseline diabetes mellitus status, and ABO blood type. *P* < 0.05 was considered statistically significant. All analyses were conducted using the R software (R Foundation for Statistical Computing, www.r-project.org, ver.4.2.2).

## Results

### Characteristics of the hospital cohort

A total of 4722 patients were included in the hospital cohort. The median age was 53 (IQR 44–60) years and 1858 (39.3%) were women. At the median follow-up time of 5 years, 819 (17.3%) had underwent DDKT. Regarding PRA, 4163 (88.2%), 412 (8.7%), and 147 (3.1%) patients belonged to the low, intermediate, and high PRA groups, respectively. Patients in the high PRA group were younger, and more likely to be women (Table 1). The high PRA group had longer waiting times and was less likely to undergo DDKT (Table 1).

**Table 1:** Clinical characteristics of the hospital cohort according to PRA groups.

Variables	Max PRA%			Total	*P*-value^a^
	< 80, N (%)	80 ≤ < 99, N (%)	≥ 99, N (%)		
Total, N (%)	4163 (88.2)	412 (8.7)	147 (3.1)	4722 (100)	< 0.001
Age, median [IQR]	53.0 [44.0–61.0]	54.0 [45.0–59.5]	50.0 [42.0–58.0]	53.0 [44.0–60.0]	0.012
Age in years, N (%)					0.064
19–40	735 (17.7)	72 (17.5)	33 (22.4)	840 (17.8)	
41–59	2221 (53.4)	237 (57.5)	84 (57.1)	2542 (53.8)	
60–	1207 (28.9)	103 (25.0)	30 (20.5)	1340 (28.4)	
Sex, N (%)					< 0.001
Male	2678 (64.3)	134 (32.5)	52 (35.4)	2864 (60.7)	
Female	1485 (35.7)	278 (67.5)	95 (64.6)	1858 (39.3)	
ABO blood type, N (%)					0.365
A or B	2555 (61.4)	250 (60.7)	81 (55.1)	2886 (61.1)	
AB	473 (10.5)	56 (13.6)	19 (12.9)	548 (11.6)	
O	1135 (28.1)	106 (25.7)	47 (32.0)	1288 (27.3)	
DM, N (%)					< 0.001
None	2614 (62.8)	298 (72.3)	113 (76.9)	3025 (64.1)	
Yes	1549 (37.2)	114 (27.7)	34 (23.1)	1697 (35.9)	
DDKT, N (%)	744 (17.9)	59 (14.3)	16 (10.9)	819 (17.3)	0.021
Follow up period, median [IQR]	5.0 [3.0–7.0]	6.0 [4.0–8.0]	7.0 [3.0–9.0]	5.0 [3.0–8.0]	< 0.001
Death while on waiting, N (%)	324 (7.8)	29 (7.0)	10 (6.8)	363 (7.7)	0.021

Patients are classified according to their maximum PRA% record.

*DDKT* deceased donor kidney transplantation, *DM* diabetes mellitus, *IQR* interquartile range, *max* maximum, *N* number, *PRA* panel reactive antibody.

^a^*P*-value for comparison among PRA groups by chi-squared test or Kruskal–Wallis test.

### Characteristics of the national cohort

Among the 18,974 patients in the national cohort, the median follow-up time was 3 years. The median age was 55 (IQR 47–62), and 39.0% were women (Supplementary Table S1). PRA was positive in 7910 (41.7%) of the patients in this cohort. Patients in the positive-PRA group were more likely to be female and less likely to undergo DDKT with longer waiting times (Supplementary Table S1).

### Impact of PRA sensitization on DDKT

**Figure 1:**
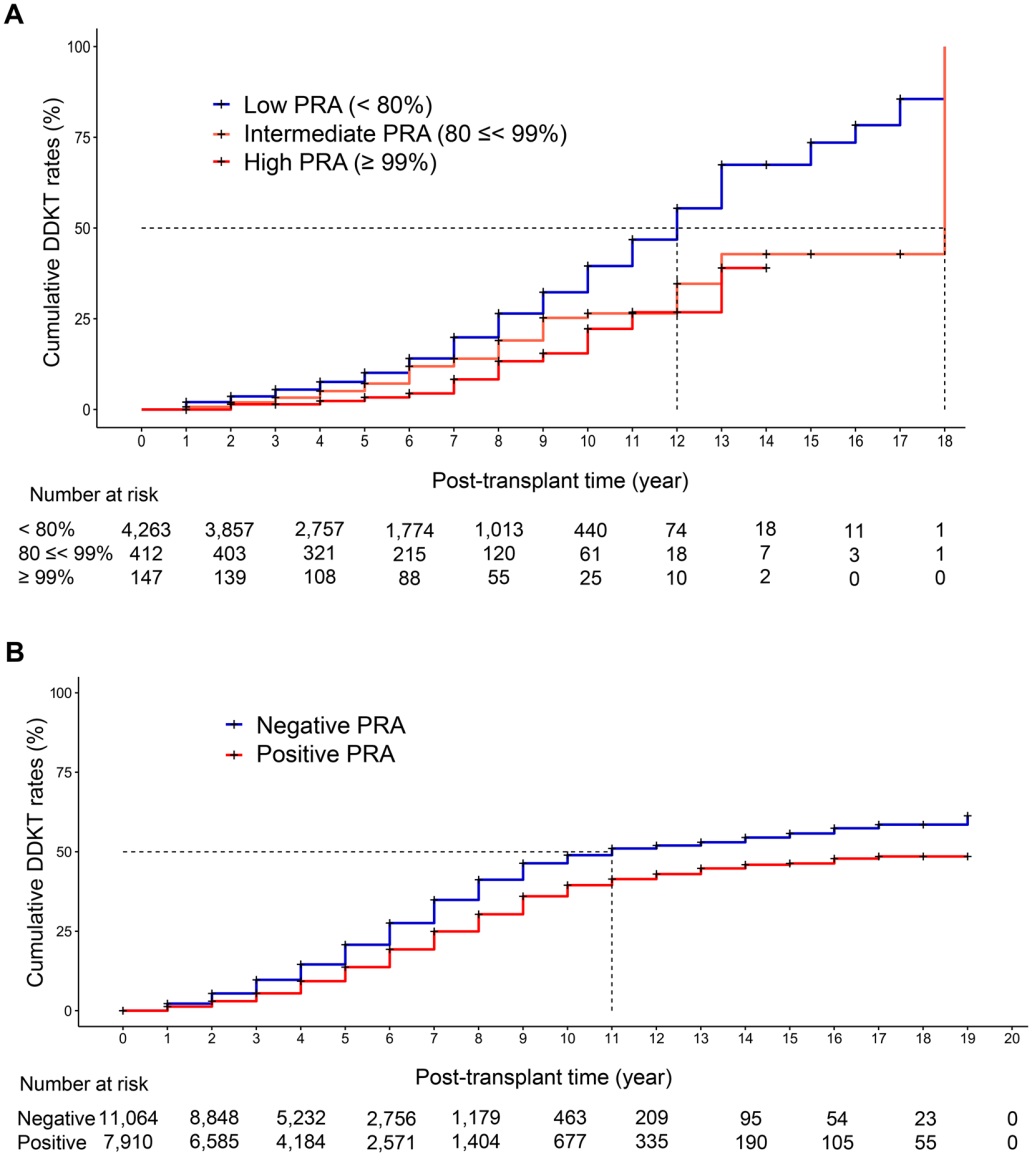
Cumulative DDKT rates according to PRA. (**A**) Cumulative DDKT rates according to PRA groups (low [< 80%], intermediate [80 ≪ 99%], high [≥ 99%]) in the hospital cohort. (**B**) Cumulative DDKT rates according to PRA groups (positive versus negative PRA) in the national cohort. *DDKT* deceased donor kidney transplantation, *max* maximum, *PRA* panel reactive antibody.

In the hospital cohort, Kaplan–Meier analyses showed that both the intermediate PRA group (80 ≤ PRA < 99%, median 18 years; 95% CI 13– not applicable) and the high PRA group (PRA ≥ 99%, median not applicable) waited longer for DDKT than the low PRA group (PRA < 80%, median 12 years; 95% CI 11–12 years) (*P* < 0.001, Fig. 1A). We found that the positive-PRA group (median 13 years; 95% CI 12–not applicable) had a longer waiting time for DDKT than the negative-PRA group (median 12 years; 95% CI 11–13 years) when PRA was categorized into negative and positive groups (*P* = 0.047).

Competing risks regression analysis revealed that the higher PRA group had a lower opportunity for DDKT after adjusting for other covariates (Table 2). Compared to the low PRA group (PRA < 80%), the intermediate PRA group (80 ≤ PRA < 99%) had a lower chance to receive DDKT by 36% (sHR, 0.64; 95% CI 0.49–0.83; *P* < 0.001) and DDKT probability was reduced by 58% (sHR, 0.42; 95% CI 0.26–0.68; *P* < 0.001) in the high PRA group (PRA ≥ 99%). When we categorized PRA into two groups, sensitized patients (positive PRA group) had fewer opportunities for DDKT (sHR, 0.85; 95% CI 0.74–0.99; *P* = 0.032) compared with the non-sensitized patients (negative PRA group). Maximal PRA% as a continuous variable was also significantly associated with a decreased likelihood of DDKT (sHR 0.99, 95% CI 0.99–1.00; *P* < 0.001).

**Table 2:** DDKT opportunity according to PRA.

Hospital cohort max PRA%	DDKT (N = 819)	Univariate model			Multivariate model		
	Yes, N (%)	sHR	95% CI	*P*-value	sHR	95% CI	*P*-value
< 80%	744 (90.8)	Reference			Reference		
80 ≪ 99%	59 (7.2)	0.66	0.51–0.86	0.002	0.64	0.49–0.83	< 0.001
≥ 99%-	16 (2.0)	0.46	0.28–0.73	0.001	0.42	0.26–0.68	< 0.001
Negative	525 (64.1)	Reference			Reference		
Positive	294 (39.5)	0.88	0.76–1.01	0.065	0.85	0.74–0.99	0.032
Max PRA% (continuous value)	819 (100.0)	0.99	0.99–1.00	< 0.001	0.99	0.99–1.00	< 0.001

National cohort PRA	DDKT (N = 3311)	Univariate model			Multivariate model		
	Yes, N (%)	sHR	95% CI	*P*-value	sHR	95% CI	*P*-value
Negative	2072 (62.6)	Reference			Reference		
Positive	1239 (37.4)	0.71	0.66–0.76	< 0.001	0.72	0.67–0.77	< 0.001

Multivariate model adjusted for age, sex, ABO blood type and DM.

Max PRA% defined as patients’ highest PRA% record.

Negative PRA indicates max PRA% was 0%

Max PRA% (cont) was treated as a continuous variable.

*CI* confidence interval, *DDKT* deceased donor kidney transplantation, *DM* diabetes mellitus, *max* maximum, *PRA* panel reactive antibody, *sHR* subdistribution hazard ratio.

Similar results were observed in a national cohort study. Kaplan–Meier analyses demonstrated that the sensitized group with positive PRA waited longer for DDKT (median not applicable years; 95% CI 16– not applicable) than the non-sensitized group with negative PRA (median 11 years; 95% CI 10–13 years) (*P* < 0.001, Fig. 1B). Competing risks regression results also showed that sensitized patients had a lower opportunity of DDKT (sHR, 0.72; 95% CI 0.67–0.77; *P* < 0.001) compared to the non-sensitized patients after adjusting for other covariates (Table 2).

### Impact of ABO blood type on DDKT

In both national and hospital cohorts, only 88.6–88.7% of type O donors were allocated to type O recipients, whereas 100.0% of type AB donors were allocated to type AB recipients and 94.5–97.4% of type A or B donors were allocated to recipients with the same blood types (Supplementary Table S2).

**Figure 2:**
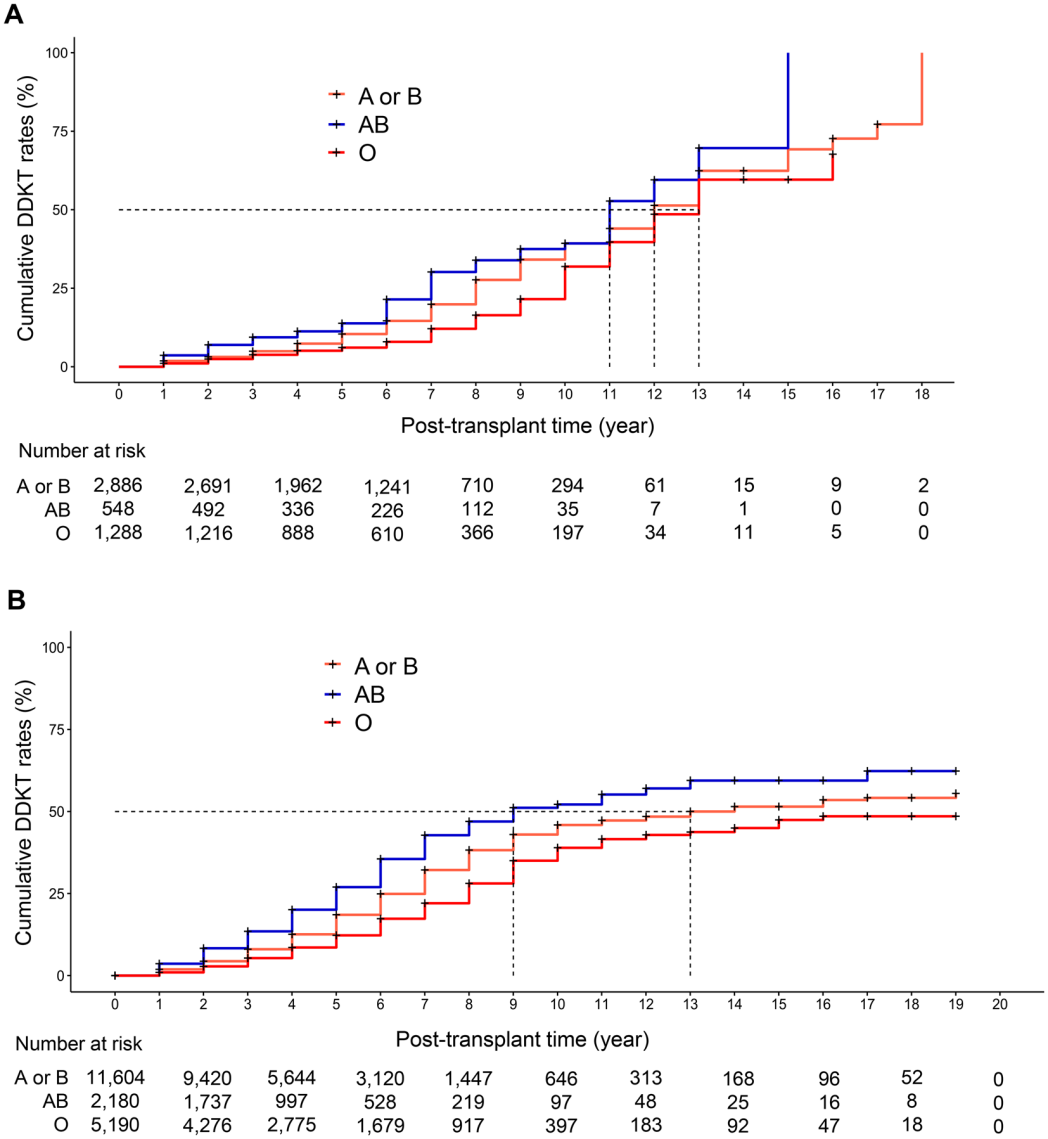
Cumulative DDKT rates according to ABO blood types in the hospital cohort (**A**) and the national cohort (**B**). *DDKT* deceased donor kidney transplantation.

In the hospital cohort, Kaplan–Meier analyses showed that the AB blood type (*P* < 0.001) waited for a shorter period and the O blood type (*P* < 0.001) waited longer for DDKT compared with the A or B blood type (Fig. 2A). Competing risks regression analysis demonstrated that blood type AB increased the probability of DDKT (sHR, 1.37; 95% CI 1.13–1.67; *P* = 0.002) and blood type O reduced DDKT probability (sHR, 0.69; 95% CI 0.59–0.82; *P* < 0.001) compared with blood types A or B (Table 3).

**Table 3:** DDKT opportunity according to ABO blood types.

	Univariate model			Multivariate model		
	sHR	95% CI	*P*-value	sHR	95% CI	*P*-value
Hospital cohort ABO blood types						
A or B	Reference			Reference		
AB	1.35	1.10–1.64	0.003	1.37	1.13–1.67	0.002
O	0.71	0.60–0.83	< 0.001	0.69	0.59–0.82	< 0.001
National cohort ABO blood types						
A or B	Reference			Reference		
AB	1.44	1.31–1.59	< 0.001	1.45	1.31–1.59	< 0.001
O	0.73	0.67–0.79	< 0.001	0.73	0.67–0.79	< 0.001

Multivariate model adjusted for age, sex, PRA, and DM.

*CI* confidence interval, *DDKT* deceased donor kidney transplantation, *DM* diabetes mellitus, *max* maximum, *PRA* panel reactive antibody, *sHR* subdistribution hazard ratio.

In the national cohort, Kaplan–Meier analyses also showed that patients with the AB blood type waited for a shorter time (*P* < 0.001) and those with the O blood type waited for a longer period (*P* < 0.001) for DDKT than those with the A or B blood type (Fig. 2B). Multivariate competing risks regression analysis demonstrated that blood type AB patients had a higher DDKT probability (sHR, 1.45; 95% CI 1.31–1.59; *P* < 0.001) and blood type O patients had a lower DDKT probability (sHR, 0.73; 95% CI 0.67–0.79; *P* < 0.001) compared with patients with blood types A or B (Table 3). Blood types A and B had similar patterns in both the hospital cohort and the national cohort and they were combined for this analysis.

### Impact of PRA sensitization and ABO blood types on DDKT in the hospital cohort

**Figure 3:**
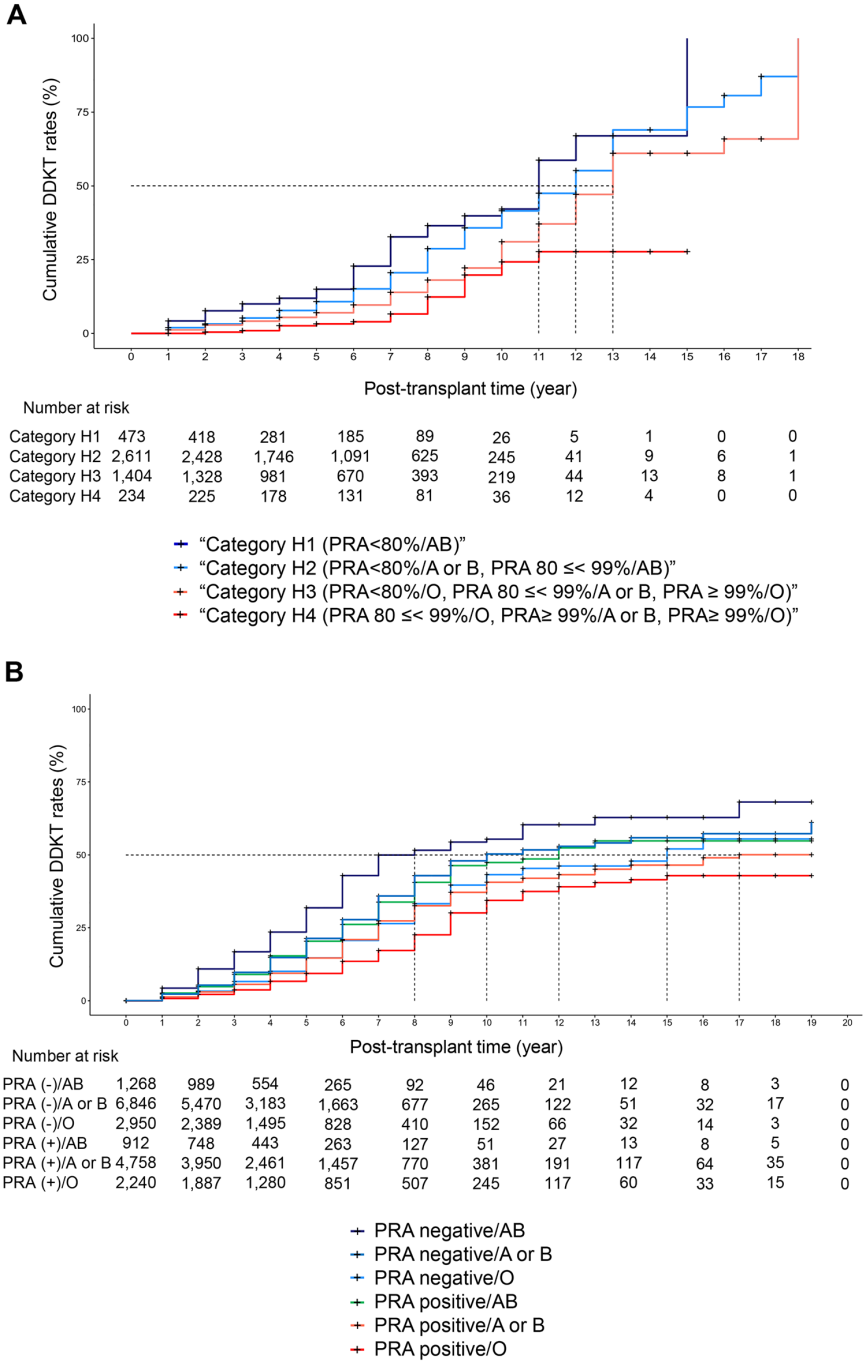
Cumulative DDKT rates according to a combination of PRA and ABO blood types. (**A**) Cumulative DDKT rates according to a combination of PRA and ABO blood types in the hospital cohort. (**B**) Cumulative DDKT rates according to a combination of PRA and ABO blood types in the national cohort. *DDKT* deceased donor kidney transplantation, *PRA* panel reactive antibody.

When we assessed DDKT accessibility according to a combination of three PRA groups (low PRA [< 80%], intermediate PRA [80 ≤  < 99%], high PRA [≥ 99%]) and three ABO blood groups (AB, A or B, O), Kaplan–Meier analyses showed that the low PRA with blood type AB had the highest DDKT opportunity (category H1, median 11 years) followed by the low PRA/A or B group and the intermediate PRA/AB group (category H2, median 12 years, *P* < 0.001, Fig. 3A). Next, the low PRA/O, intermediate PRA/A or B, and high PRA/AB groups (category H3, median 13 years) had fewer opportunities to access DDKT than category H2 (*P* < 0.001, Fig. 3A). The intermediate PRA/O and high PRA/A, B, or O groups had the lowest DDKT opportunity (category H4, median not applicable, *P* = 0.001 compared to category H3, Fig. 3A).

Next, we compared DDKT opportunity according to the combination of the three PRA and three ABO blood groups using competing risks regression analysis (Table 4). Compared with the low PRA/AB group (reference group), the low PRA/A or B groups (sHR, 0.72) had fewer opportunities. The low PRA/O group (sHR, 0.50), intermediate PRA/A or B group (sHR, 0.48), and the high PRA/AB group (sHR, 0.35) also had fewer opportunities than the low PRA/AB group. Furthermore, the intermediate PRA/O group (sHR, 0.29), high PRA/A or B group (sHR, 0.26), and high PRA/O group had the lowest opportunities (sHR, 0.31).

**Table 4:** DDKT opportunity according to combination of PRA and ABO blood types.

		ABO blood types					
		AB	*P*-value	A or B	*P*-value	O	*P*-value
Hospital cohort sHR (95% CI)							
Max PRA%	< 80%	Reference		0.72 (0.58–0.88)	0.002	0.50 (0.39–0.63)	< 0.001
	80 ≪ 99%	0.56 (0.28–1.12)	0.100	0.48 (0.33–0.69)	< 0.001	0.29 (0.15–0.55)	< 0.001
	≥ 99%-	0.35 (0.12–1.03)	0.057	0.26 (0.13–0.54)	< 0.001	0.31 (0.14–0.70)	0.005
National cohort sHR (95% CI)							
PRA	Negative	Reference		0.66 (0.59–0.75)	< 0.001	0.47 (0.41–0.55)	< 0.001
	Positive	0.65 (0.54–0.78)	< 0.001	0.48 (0.42–0.55)	< 0.001	0.36 (0.31–0.42)	< 0.001

Multivariate model adjusted for age, sex, and DM.

Max PRA% defined as patients’ highest PRA% record.

*CI* confidence interval, *DDKT* deceased donor kidney transplantation, *DM* diabetes mellitus, *max* maximum, *PRA* panel reactive antibody, *sHR* subdistribution hazard ratio.

When groups with similar DDKT opportunities were divided into four categories, as shown in Fig. 3A, we observed a categorical hierarchy of DDKT opportunities as follows: 1 (reference), 2 (sHR 0.71), 3 (sHR 0.49), and 4 (sHR 0.28) in the multivariate analysis (Table 5).

**Table 5:** DDKT opportunity according to category based on PRA and ABO blood types.

	Median waiting time	Univariate model			Multivariate model		
		sHR	95% CI	*P*-value	sHR	95% CI	*P*-value
Hospital cohort Category H							
PRA < 80%/AB (category H1)	11	Reference			Reference		
PRA < 80%/A or B, PRA 80 ≪ 99%/AB (category H2)	12	0.71	0.58–0.88	0.001	0.71	0.58–0.88	0.001
PRA < 80%/O, PRA 80 ≪ 99%/A or B, PRA ≥ 99%/AB (category H3)	13	0.50	0.40–0.63	< 0.001	0.49	0.39–0.62	< 0.001
PRA 80 ≪ 99%/O, PRA ≥ 99%/A or B, PRA ≥ 99%/O (category H4)	NA	0.30	0.19–0.46	< 0.001	0.28	0.18–0.44	< 0.001
National cohort Category N							
PRA negative/AB (category N1)	8	Reference			Reference		
PRA negative/A or B, PRA positive/AB (category N2)	11	0.66	0.59–0.75	< 0.001	0.66	0.59–0.75	< 0.001
PRA negative/O, PRA positive/A or B (category N3)	16	0.48	0.42–0.54	< 0.001	0.48	0.42–0.54	< 0.001
PRA positive/O (category N4)	NA	0.36	0.31–0.41	< 0.001	0.36	0.31–0.42	< 0.001

Multivariate model adjusted for age, sex, and DM. Max PRA% defined as patients’ highest PRA% record.

*CI* confidence interval, *DDKT* deceased donor kidney transplantation, *DM* diabetes mellitus, *max* maximum, *PRA* panel reactive antibody, *sHR* subdistribution hazard ratio.

### Impact of PRA sensitization and ABO blood types on DDKT in the national cohort

In the national cohort, we compared DDKT accessibility according to the combination of the two PRA groups and three ABO blood types (Table 4, Fig. 3B). Kaplan–Meier analyses showed that the negative PRA/A or B group (median, 10 years) and positive PRA/AB group (median 12 years) waited longer for DDKT than the negative PRA/AB group (median 8 years, *P* < 0.001, Fig. 3B). The negative PRA/O group (median, 15 years) and the positive PRA/A or B groups (median, 17 years) waited for a much longer time for DDKT. Moreover, the positive PRA/O group waited for the longest (median, not applicable, Fig. 3B).

In parallel with the survival curves, multivariate competing risks regression analysis demonstrated a similar hierarchy in DDKT opportunity according to the combination of PRA and ABO blood types (Table 4). Compared to the negative PRA/AB group (reference group), the negative PRA/A or B group (sHR, 0.66) and positive PRA/AB group (sHR, 0.65) had fewer opportunities. The negative PRA/O group (sHR, 0.47) and the positive PRA/A or B groups (sHR, 0.48) had much fewer opportunities. Furthermore, the positive PRA/O group had the lowest number of opportunities (sHR, 0.36).

When groups with similar DDKT opportunities were divided into four categories, there was a categorical hierarchy of DDKT opportunities in the following order: 1 (median, 8 years), 2 (median, 11 years, sHR 0.66), 3 (median, 16 years, sHR 0.48), and 4 (median not applicable, sHR 0.36) in the multivariate analysis (Table 5).

## Discussion

This study found that sensitization, represented by a positive or high PRA, increased the waiting period for DDKT and decreased DDKT opportunities in both hospital-based and national cohorts. Additionally, the waiting time or accessibility to DDKT differed according to the ABO blood type. Furthermore, by categorizing waitlisted patients according to a combination of PRA and ABO blood types, we identified a more precise hierarchy of DDKT opportunities.

Previous studies in the USA, Australia, and Mexico have shown that the probability of receiving DDKT decreases with higher PRA levels^17–19^. A German study reported a decreased likelihood of receiving DDKT for candidates with a virtual PRA (vPRA) > 85% who are younger than 65 years, and those with a vPRA > 50% who are older than 65 years. In both age groups, patients with a vPRA > 95% showed a further decrease in DDKT probability and longer waiting times^9^. According to the current Korean kidney allocation system, we also found that the probability of receiving DDKT decreases with higher PRA levels. Positive PRA significantly decreased the opportunity for DDKT in both hospital-based and national cohorts. Furthermore, both the high and the intermediate PRA groups had fewer DDKT opportunities than the low PRA group in the hospital cohort. Since the median DDKT waiting time is > 10 years in Korea, even for non-sensitized waitlisted patients, the delay due to sensitization probably has a more serious impact on DDKT opportunity for highly-sensitized patients, particularly in Korea compared with Western countries.

To resolve the sensitization-related inequity in DDKT opportunity, a new kidney allocation system was introduced in the US in 2014, which utilizes a sliding scale ranging from 0 to 200 points for candidates based on cPRA levels instead of the previous low scores (0–4)^11,20^. After the implementation of the new allocation system, particularly for highly-sensitized patients with a cPRA ≥ 90%, there have been improvements in the kidney access disparity related to HLA sensitization without any difference in graft survival outcomes^21,22^. In Israel, the adoption of the sliding scale points based on PRA improved equity in highly-sensitized patients. In the UK, Australia, and Eurotransplant, kidney allocation systems proved additional points for highly-sensitized candidates based on PRA to compensate for sensitization-related disparity^12^.

Our study revealed a significant difference in DDKT opportunities among different ABO blood types in both hospital-based and national cohorts. Patients with blood type O had the lowest DDKT opportunity, whereas those with blood types A or B had an intermediate DDKT opportunity, and those with blood type AB had the highest DDKT opportunity. Blood type O candidates received less than half of the opportunity for DDKT compared with blood type AB candidates and had more than 30% fewer chances for DDKT compared with non-O blood type candidates. This disparity occurred because candidates with O type are restricted to ABO-identical transplantation, whereas kidneys from blood group O donors can be given to non-O blood group recipients if no candidate with the same ABO blood type is available. Despite the disparity in DDKT opportunities due to ABO blood type, most of kidney allocation systems do not address this including those in the US, UK, Eurotransplant, France, and Korea^10–12,23^. Therefore, additional points should be considered for candidates with disadvantaged blood types and high PRA to correct the current inequity.

Efforts to reduce the inequity related to ABO blood type have been attempted for the A2 subtype of blood type A^24–26^. Since the antigen expression of A2 subtype is much lower than that of A1, A2 is functionally similar to blood type O and A2B is similar to blood type B in terms of ABO antigen expression^27^. In the US, access to transplantation for blood type B was increased to improve equity. Owing to the prolonged waiting time among other blood groups, the new 2014 KAS preferentially allocated A2 and A2B kidneys to B candidates^28,29^. Since the introduction of the new KAS, utilization of A2 kidneys for blood group B candidates has increased, while patient and graft outcomes remained consistent with those of conventional ABO-compatible DDKT, although there is a report of increased anti-A titer^30–33^. In a recent Canadian study, a new ABO-adjusted cPRA, which adjusts ABO sensitization on the same scale as HLA sensitization, was proposed to solve the disparity in kidney allocation for blood types B and O. Similarly, for the calculation of cPRA based on HLA sensitization, the ABO-adjusted cPRA is calculated based on the frequency of ABO blood groups in the donor pools. Candidates with blood types B and O, who have fewer opportunities for DDKT due to their blood type, can be assigned more bonus kidney allocation points through this system^34,35^.

We found that both the PRA and ABO blood types had significant effects on DDKT accessibility. Therefore, we assessed DDKT accessibility according to a combination of PRA status and ABO blood type. In the hospital-based cohort with more detailed PRA information, we categorized the groups into four categories with similar DDKT opportunities. First, the low PRA with blood type AB group (category H1) had the highest probability of receiving DDKT, followed by the low PRA/A or B and the intermediate PRA/AB groups (category H2). Next, the low PRA/O, intermediate PRA/A or B, and high PRA/AB groups (category H3) had much lower DDKT opportunities. The intermediate PRA/O and high PRA/non-AB groups (category H4) had the lowest probability of DDKT. In the national cohort with limited PRA information, we also categorized the groups into four categories with similar DDKT opportunities. Negative PRA with blood AB (category N1) had the highest DDKT opportunity, while positive PRA with blood group O (category N4) had the lowest DDKT opportunity. These data suggest that the combination of PRA and ABO blood type could provide more precise information regarding DDKT accessibility to waitlisted patients and medical staff than PRA information alone.

Since sensitization and ABO blood type significantly increased the waiting time for DDKT, priority scores should be given to sensitized candidates with blood type O to solve the inequity in DDKT opportunities. This issue is more critical in Asian countries, including Korea, which have long waiting times. For example, the recent mean waiting time was 6.5 years among Korean DDKT patients^36^. Moreover, the median waiting time until DDKT is > 10 years in Korea if all waitlisted patients are included in the calculation. However, the current Korean allocation system coordinated by KONOS, a governmental agency, only assigns two additional points to patients with prior cross-match positivity or a history of prior transplantation, and no additional points according to ABO blood type^37^. Given that the waiting time for highly sensitized patients has significantly decreased after the introduction of a new kidney allocation system in the US^21,38^, we need to revise the current system with higher compensatory points for waitlisted patients with a high PRA or blood type O to reduce the disadvantage of this population. Considering that the mean waiting time for DDKT patients ≤ 18 years who receive additional 3–4 points depending on age is 2.5 years, in contrast to adult DDKT patients with a median waiting time of 6.5 years, the appropriate advantage points could effectively improve the existing inequities related to blood types and PRA in the organ allocation process in Korea^36^.

To assign higher points based on PRA and ABO blood types for equitable kidney allocation, we also need to verify whether or not such allocation systems result in worse patient and graft survival on a national scale with respect to utility. For example, the allocation of more kidneys to sensitized patients without donor-specific antibodies would have a potential risk of increased rejection and worse graft outcomes. In addition to more benefits to highly-sensitized patients and patients with A2 blood type, the new US KAS has not led to worse graft and patient outcomes to date^21,22,30–33^. However, the outcomes for very highly sensitized patients with cPRA 100% is still controversial^38^. Therefore, a new kidney allocation scoring system that balances equity with utility should be developed. Even after the introduction of the new allocation system, a follow-up assessment is essential to determine whether assigning additional allocation points based on PRA and blood type would significantly enhance equity without compromising utility.

This study had several limitations. First, our cohorts did not implement cPRA, because cPRA had not been used in most Korean centers and it was recently introduced. We used the max PRA% for the analysis, which represented the highest PRA values between PRA Class I and II. This could have led to an overestimation of PRA values; therefore, a direct comparison with other kidney allocation systems that use cPRA may be difficult. Second, the impact of PRA% was analyzed only in the hospital cohort, and the results may not be representative of the entire Korean DDKT population because the national cohort provided only PRA positivity. Further studies using cPRA and ABO blood types are required to confirm these findings.

Nevertheless, this is the first nationwide study on the impact of sensitization on DDKT opportunity in Asian countries with prolonged waiting time and is expected to contribute to a better understanding of the current allocation system beyond Western countries. Furthermore, the analysis of the impact of PRA in combination with ABO blood type is a new approach solving inequity according to ABO blood type as well as PRA. We hope to develop a more integrated allocation scoring system based on this approach.

In summary, we found that both PRA and ABO blood types had significant impacts on DDKT opportunities. Highly-sensitized waitlisted patients with blood type O are unfairly disadvantaged, leading to serious inequity, especially in Asian countries with very long waiting times. Therefore, a new allocation system with higher additional points based on PRA and ABO blood types is required to improve the inequity in DDKT opportunities.

### Supplementary Information

Supplementary Information.

## Data Availability

Data supporting the findings of this study are available from the corresponding author upon reasonable request.
