# Supplementary material for: Impact of sensitization and ABO blood types on the opportunity of deceased-donor kidney transplantation with prolonged waiting time
Source: Sci Rep. 2024 Feb 1;14:2635. doi: 10.1038/s41598-024-53157-2 (PMC10834527; doi:10.1038/s41598-024-53157-2)
Supplement: Supplementary file 1 — Supplementary Information. [file 41598_2024_53157_MOESM1_ESM.docx]

**SUPPLEMENTARY MATERIALS**

**Impact of sensitization and ABO blood types on the opportunity of deceased-donor kidney transplantation with prolonged waiting time**

Correspondence: Jaeseok Yang (jcyjs@yuhs.ac)

**CONTENTS**

**Supplementary Table S1**. Clinical characteristics of the national cohort according to PRA groups

**Supplementary Table S2**. The distribution of ABO blood types in recipeints and donors.

**Supplementary Table S3**. Clinical characteristics of the national cohort excluding the hospital cohorts (new cohort) according to PRA groups.

**Supplementary Table S4.** DDKT opportunity according to PRA and ABO blood types in the new cohort.

**Supplementary Table S5.** DDKT opportunity according to combination of PRA and ABO blood types in the new cohort.

**Supplementary Figure S1.** Flow diagram of the study.

**Supplementary Table S1**. Clinical characteristics of the national cohort according to PRA groups.

| Variables | **PRA** | | Total | *P*-value^a^ |
| --- | --- | --- | --- | --- |
|  | **Negative** | **Positive** |  |  |
| Total, N (%) | 11,064 (58.3) | 7,910 (41.7) | 18,974 (100.0) |  |
| Age, median [IQR] | 55.0 [46.0-62.0] | 55.0 [47.0-61.0] | 55.0 [47.0-62.0] | 0.331 |
| Age in years, N (%) |  |  |  | <0.001 |
| 19-40 | 1,493 (13.5) | 855 (10.8) | 2,348 (12.4) |  |
| 41-59 | 5,959 (53.9) | 4,526 (57.2) | 10,385 (55.3) |  |
| 60- | 3,612 (32.6) | 2,529 (32.0) | 6,141 (32.4) |  |
| Sex, N (%) |  |  |  | <0.001 |
| Male | 8,128 (73.5) | 3,448 (43.6) | 11,576 (61.0) |  |
| Female | 2,936 (26.5) | 4,462 (56.4) | 7,398 (39.0) |  |
| ABO blood type, N (%) |  |  |  | 0.032 |
| A or B | 6,846 (61.9) | 4,758 (60.2) | 11,604 (61.2) |  |
| AB | 1,268 (11.5) | 912 (11.5) | 2,180 (11.5) |  |
| O | 2,950 (26.6) | 2,240 (28.3) | 5,190 (27.4) |  |
| Follow up period,  median [IQR] | 3.0 [2.0-5.0] | 4.0 [2.0-6.0] | 3.0 [2.0-6.0] | <0.001 |
| DDKT, N (%) | 2,072 (18.7) | 1,239 (15.7) | 3,311 (17.5) | <0.001 |
| Death while on waiting,  N (%) | 1,068 (9.7) | 756 (9.6) | 1,824 (9.6) | <0.001 |

Patients are classified according to their PRA.

Abbreviations: DDKT, deceased donor kidney transplantation; PRA, panel reactive antibody.

^a^*P*-value for comparison among PRA groups by chi-squared test or Mann-Whitney test.

**Supplementary Table S2**. The distribution of ABO blood types in recipeints and donors.

| **Hospital cohort** | | Recipient, N (%) | | | |  |
| --- | --- | --- | --- | --- | --- | --- |
|  |  | A | B | AB | O | Total |
| Donor, N (%) | A | 277 (94.5) | 0 (0.0) | 16 (5.5) | 0 (0.0) | 293 (35.8) |
|  | B | 0 (0.0) | 224 (97.4) | 6 (2.6) | 0 (0.0) | 230 (28.1) |
|  | AB | 0 (0.0) | 0 (0.0) | 92 (100.0) | 0 (0.0) | 92 (11.2) |
|  | O | 8 (3.9) | 8 (3.9) | 7 (3.4) | 181 (88.7) | 204 (24.9) |
|  | Total | 286 (34.9) | 232 (28.3) | 120 (14.7) | 181 (22.1) | 819 (100.0) |
| **National cohort** | | Recipient, N (%) | | | |  |
|  |  | A | B | AB | O | Total |
| Donor, N (%) | A | 1,113 (96.7) | 0 (0.0) | 38 (3.3) | 0 (0.0) | 1,151 (34.8) |
|  | B | 0 (0.0) | 855 (96.5) | 31 (3.5) | 0 (0.0) | 886 (26.8) |
|  | AB | 0 (0.0) | 0 (0.0) | 429 (100.0) | 0 (0.0) | 429 (13.0) |
|  | O | 38 (4.5) | 37 (4.4) | 21 (2.5) | 749 (88.6) | 845 (26.5) |
|  | Total | 1,151 (34.8) | 892 (26.9) | 519 (15.7) | 749 (22.6) | 3,311 (100.0) |

**Supplementary Table S3.** Clinical characteristics of the national cohort excluding the hospital cohorts (new cohort) according to PRA groups.

| Variables | PRA | | Total | *P*-value^a^ |
| --- | --- | --- | --- | --- |
|  | Negative | Positive |  |  |
| Total, N (%) | 8,332 (58.5) | 5,920 (41.5) | 14,252 (100.0) |  |
| Age, median [IQR] | 55.0 [46.0-61.0] | 54.0 [47.0-61.0] | 55.0 [47.0-61.0] | 0.462 |
| Age in years, N (%) |  |  |  | <0.001 |
| 19-40 | 1,102 (13.2) | 618 (10.4) | 1,720 (12.1) |  |
| 41-59 | 4,590 (55.1) | 3,488 (58.9) | 8,078 (56.7) |  |
| 60- | 2,640 (31.7) | 1,814 (30.6) | 4,454 (31.3) |  |
| Sex, N (%) |  |  |  | <0.001 |
| Male | 6,125 (73.5) | 2,580 (43.6) | 8,705 (61.1) |  |
| Female | 2,207 (26.5) | 3,340 (56.4) | 5,547 (38.9) |  |
| ABO blood type, N (%) |  |  |  | 0.013 |
| A or B | 5,199 (62.4) | 3,578 (60.4) | 8,777 (61.6) |  |
| AB | 957 (11.5) | 665 (11.2) | 1,622 (11.4) |  |
| O | 2,176 (26.1) | 1,677 (28.3) | 3,853 (27.0) |  |
| Follow up period,  median [IQR] | 3.0 [2.0-5.0] | 4.0 [2.0-6.0] | 3.0 [2.0-6.0] | <0.001 |
| DDKT, N (%) | 1,645 (19.7) | 986 (16.7) | 2,631 (18.5) | <0.001 |
| Death while on waiting, N (%) | 813 (9.8) | 558 (9.4) | 1,371 (9.6) | <0.001 |

Patients are classified according to their PRA.

Abbreviations: DDKT, deceased donor kidney transplantation; PRA, panel reactive antibody.

^a^*P*-value for comparison among PRA groups by chi-squared test or Mann-Whitney test.

**Supplementary Table S4.** DDKT opportunity according to PRA and ABO blood types in the new cohort.

| **New cohort**  **PRA** | | Univariate model | | | Multivariate model | | |
| --- | --- | --- | --- | --- | --- | --- | --- |
|  |  | sHR | 95% CI | *P*-value | sHR | 95% CI | *P*-value |
| Negative | | Reference |  |  | Reference |  |  |
| Positive | | 0.73 | 0.68-0.79 | <0.001 | 0.75 | 0.69-0.81 | <0.001 |
| **New cohort**  **ABO types** | | Univariate model | | | Multivariate model | | |
|  |  | sHR | 95% CI | *P*-value | sHR | 95% CI | *P*-value |
| A or B | | Reference |  |  | Reference |  |  |
| AB | | 1.43 | 1.29-1.60 | <0.001 | 1.43 | 1.29-1.59 | <0.001 |
| O | | 0.73 | 0.66-0.79 | <0.001 | 0.73 | 0.66-0.79 | <0.001 |
| **New cohort**  **sHR (95% CI)** | | ABO blood types | | | | | |
|  |  | **AB** | *P*-value | **A or B** | *P*-value | **O** | *P*-value |
| **PRA** | Negative | Reference |  | 0.68 (0.59-0.78) | <0.001 | 0.49 (0.42-0.57) | <0.001 |
|  | Positive | 0.70 (0.57-0.86) | <0.001 | 0.51 (0.44-0.59) | <0.001 | 0.37 (0.32-0.45) | <0.001 |

Multivariate model adjusted for age, sex, ABO blood type and PRA.

Negative PRA indicates max PRA% was 0%.

Abbreviations: CI, confidence interval; DDKT, deceased donor kidney transplantation; PRA, panel reactive antibody; sHR, subdistribution hazard ratio.

**Supplementary Table S5.** DDKT opportunity according to combination of PRA and ABO blood types in the new cohort.

| **New cohort**  **Category N** | Median waiting time | Univariate model | | | Multivariate model | | |
| --- | --- | --- | --- | --- | --- | --- | --- |
|  |  | sHR | 95% CI | *P*-value | sHR | 95% CI | *P*-value |
| PRA negative/AB  (category N1) | 7 | Reference |  |  | Reference |  |  |
| PRA negative/A or B,  PRA positive/AB  (category N2) | 9 | 0.69 | 0.60-0.79 | <0.001 | 0.68 | 0.60-0.78 | <0.001 |
| PRA negative/O,  PRA positive/A or B  (category N3) | 12 | 0.51 | 0.45-0.59 | <0.001 | 0.50 | 0.44-0.58 | <0.001 |
| PRA positive/O  (category N4) | NA | 0.37 | 0.31-0.44 | <0.001 | 0.37 | 0.31-0.44 | <0.001 |

Multivariate model adjusted for age, and sex.

Abbreviations: CI, confidence interval; DDKT, deceased donor kidney transplantation; PRA, panel reactive antibody; sHR, subdistribution hazard ratio.

**

**

**Supplementary Figure S1.** Flow diagram of the study.

Abbreviations: DDKT, deceased donor kidney transplantation; PRA, panel reactive antibody.
